# A few long versus many short foraging trips: different foraging strategies of lesser kestrel sexes during breeding

**DOI:** 10.1186/s40462-017-0100-6

**Published:** 2017-04-25

**Authors:** Jesús Hernández-Pliego, Carlos Rodríguez, Javier Bustamante

**Affiliations:** 1 Department of Wetland Ecology, Estación Biológica de Doñana (EBD-CSIC), c/Américo Vespucio s/n, 41092 Seville, Spain; 2 Department of Conservation Biology, Estación Biológica de Doñana (EBD-CSIC), c/Américo Vespucio s/n, 41092 Seville, Spain

**Keywords:** *Falco naumanni*, Role specialization, Foraging behavior, Spatial segregation, Movement ecology, Breeding ecology, GPS, Biologging

## Abstract

**Background:**

In species with biparental care both members of the breeding pair cooperate to raise the offspring either by assisting each other in every reproductive task or by specializing in different ones. The latter case is known as reproductive role specialization. Raptors are considered one of the most role-specialized groups, but little is known about parental behavior away from the nest. Until the advent of biologgers, avian role specialization was traditionally studied with direct observations at the nest because of the difficulties of following and recording the behavior of free-ranging individuals. In this paper we analyze how the role specialization of the lesser kestrel (*Falco naumanni*) influences foraging movement patterns throughout the breeding season. We tracked 30 lesser kestrel breeders from two breeding colonies using high-frequency GPS-dataloggers during four consecutive breeding seasons.

**Results:**

We found no differences between sexes in lesser kestrel foraging movements early in the breeding season before the formation of the breeding pair. However, we observed sexually distinct foraging movement strategies later in the breeding season once breeding pairs were formed. Lesser kestrel males performed a large number of short foraging trips while females made a few long ones. This maximized the provisioning rate by males to feed their mates and offspring. Meanwhile, lesser kestrel females spent more time at the colony than males in order to defend the nest, incubate the eggs and brood the nestlings. Females also helped their mates to provision the nestling once these had grown and required more food and less protection. Furthermore, lesser kestrels showed a sexual spatial segregation in foraging areas, with males foraging closer to the colony than females.

**Conclusions:**

The lesser kestrel responds to changes in energy demand throughout the breeding season with its foraging movement strategy, but in a different way depending on parental sex. The sexual spatial segregation observed is likely to be the result of an adaptive foraging strategy based on role specialization to reduce prey depletion close to the colony and intersexual competition in order to improve breeding success.

**Electronic supplementary material:**

The online version of this article (doi:10.1186/s40462-017-0100-6) contains supplementary material, which is available to authorized users.

## Background

Parental care includes any behavior in adult breeders that results in promoting offspring survival at the cost of compromising their own survival and future reproductions, because of the energy and time invested [1, 2]. Biparental care entails that both members of the breeding pair are involved in raising the offspring [3]. Indeed, cooperation between parents is essential for successful breeding as it takes care of several reproductive tasks, such as the construction of nests, the incubation of eggs or the provisioning of the offspring (see [4]). In some species both members of the breeding pair cooperate by assisting their partner in every reproductive task, whereas in other species one of the parents specializes in a number of specific tasks. The latter case is known as reproductive role specialization [5]. Thus, the females of a role-specialized species assume certain reproductive tasks while the males are responsible for others, thereby balancing parental investment throughout the breeding season. Role specialization has predominantly been studied in birds, a factor that is probably due to the high percentage of species (>80%) with biparental care [6, 7], although role specialization has also been described in insects, fishes, and mammals [8–10]. Literature on avian role specialization has been traditionally based on direct observation at the nest, with no considerations on parental behavior away from it because of the difficulty of tracking mobile individuals across the landscape (e.g., [11–13]). However, new biologging technologies are providing us with new tools to monitor animal behavior remotely [14, 15].

The revolution in animal tracking systems has led to a rapid expansion of movement ecology as a new discipline whose primary objective is to create a conceptual framework to unify the study of movement [16]. According to this paradigm, individual movement results from the interaction of four elements: external agents, motion abilities, navigation capacities, and internal state [17]. In role-specialized species, each member of the breeding pair performs specific reproductive tasks throughout the breeding season; as a result parental sex is expected to exert a strong influence on movement behavior in order to satisfy the temporally dynamic requirements during reproduction. Indeed, sexually distinct movement patterns have been attributed to role specialization of sexes in several species of seabirds (e.g., [18–20]). Nevertheless, virtually nothing is known about how role specialization influences parental movements in raptors, even though this group is among the most role-specialized of birds [21, 22]. Role specialization is well documented in raptors thanks to direct observation at the nest: males are mainly responsible for provisioning tasks to feed their mate or chicks, whereas females take care of nest defense, egg incubation and chick brooding [23–26]. In this paper we investigate the effect of role specialization on the foraging movement behavior of the lesser kestrel (*Falco naumanni*) during the breeding season.

The lesser kestrel is an small insectivorous raptor that winters in Africa and breeds across the Palearctic [27]. This species presents reversed sexual size dimorphism with females being larger than males (~15% difference in body mass), which is common among raptor species [22]. It also presents a strong sexual chromatic dimorphism in plumage (males have a blue-gray plumage in their head and tail, whereas females have a uniform rusty plumage with black stripes) [27]. Lesser kestrels are colonial breeders nesting in holes in buildings and cliffs in steppe-like habitats or non-irrigated arable crops in western Europe [28]. Lesser kestrels behave as central-place foragers during breeding. They fly from the colony to a foraging patch where they capture prey and return to the colony carrying a single prey item in their beak or talons [29]. Kestrels can capture prey either by active hovering flights or with a sit-and-wait strategy from a perch [30]. Although commonly considered flapping raptors, it has recently been shown that lesser kestrels frequently use thermal soaring to commute to the foraging patch [31, 32]. The role specialization of the lesser kestrel has been studied through direct observations at the nest and it matches the general trend of task division in raptors [33–36]. Males provide food to their mates before egg-laying and during incubation and assume a dominant role during the feeding of nestlings. Females do a larger share of the incubation and brood recently hatched chicks. In this study, we tracked individual lesser kestrels using high-frequency GPS-dataloggers to study foraging movement behavior throughout the breeding season. Kestrel breeders are expected to respond to changes in energy demand along the different phenological periods into which the breeding season can be divided with a different movement strategy according to sex. We hypothesized that, since the lesser kestrel is a role-specialized species, (1) both sexes will exhibit a different movement strategy likely to be reflected in variables like accumulated distance, number of foraging trips, colony attendance, or foraging trip duration. Differences in movement behavior could also be attributed to sexual dimorphism in size or color. If this were the case (2) we would expect that differences in movement between the sexes would remain constant along the breeding season because dimorphism in size or color does not change. If role-specialization is the main driver for a different movement strategy, (3) differences will be minor during the periods in which both sexes perform similar roles and will be more pronounced when roles differ the most.

After spring migration, lesser kestrels arrive at the breeding colony where they start to select mate and a nest hole. In this establishment period, the breeding pair is still unformed, and consequently there is no role specialization. In this period, we would expect (4) no sexual differences in lesser kestrel foraging movement variables like daily distance traveled, number of foraging trips, colony attendance, or foraging trip duration. Once the breeding pairs are formed and nest have been selected (courtship, incubation and nestling periods), we would expect that (5) lesser kestrel males would perform a higher number of foraging trips per day than females, as the main sex responsible for provisioning tasks; and (6) females would stay longer than males at the colony in order to defend the nest, incubate eggs and/or brood chicks. We would expect that (7) both sexes would increase the distance traveled and the number of foraging trips per day, and they would also decrease daily colony attendance, as parental investment increases from the establishment to nestling period. (8) This increase should be most notable along the nestling period when chick growth increases parental investment (see [37]).

We also analyzed the temporal evolution of adult body mass as an indicator of individual condition that is expected to be inversely related to parental investment throughout the breeding season. In addition, we evaluated sexual differences in habitat selection, hunting strategy and foraging areas throughout the breeding season as alternative explanations for some of the differences observed in movement strategy.

## Methods

### Study area

We studied lesser kestrels from two breeding colonies located in the Guadalquivir river basin (southwestern Spain), which is dominated by arable crops [38]. Wheat and sunflower are the primary crops at the study area, although olives and vineyards are also present. The Silo colony is situated at a building with a grain elevator and is surrounded by an agricultural landscape in La Palma del Condado (Huelva, Andalusia), whereas the EBD colony, on the roof of our research institute, is surrounded by the mainly urban landscape of the city of Seville (Andalusia). Lesser kestrel pairs breed inside nest-boxes installed at both buildings.

### Instrumentation and fieldwork

Lesser kestrel breeding pairs were monitored during 4 consecutive breeding seasons (years 2011–2014). We tracked individual lesser kestrels using GPS-dataloggers (GiPSy models 2, 4, and 5; weighing up to 2 g; Technosmart, Rome, Italy) with small-sized batteries (90–100 mA, 2.2 g). GPS were fixed to the birds’ backs using a micro back-pack harness from Marshall Radio Telemetry (North Salt Lake, Utah, U.S.A.) or a similar hand-made harness formed by a carbon fiber plate and a 4 mm width teflon ribbon (Bally Ribbon Mills, Pennsylvania, U.S.A.). GPS-dataloggers were covered with a protective thermoretractable case. The total mass of the equipment (harness + GPS + battery) was about 6 g and never exceeded the 5% of lesser kestrel mean body mass (130 g, e.g. [27]), which is within the recommended limits for flying animals [39]. To accustom the birds to the harness and the GPS device, we fixed a dummy GPS-datalogger with the same weight to the harness at least a week before fixing the real device and recording the birds’ movement (see details of the procedure in [40]).

We obtained a total of 825,365 fixes from 35 individuals (a mean ± standard deviation of 23,581.86 ± 16,113.46 fixes per individual, range 3275–55,273). Some of them were tracked during two (8 individuals) or three (1 individual) breeding seasons. Nevertheless, 5 kestrels finally did not breed at the study colonies and their data were excluded from the analyses. Statistical analyses were performed using tracking data from 30 lesser kestrel breeders (14 females and 16 males). We configured GPS devices at one of five different sampling frequencies: one fix every second, one fix every minute or one fix every 3, 5, and 10 min. We recaptured tracked kestrels to recover the data stored in the logger. A new full-powered GPS device was then deployed before releasing the individual to resume tracking. Kestrels were captured when they entered nest-boxes using remote-controlled sliding doors. Individuals were captured a mean of 7.63 ± 2.46 times a year, range 2–11 (*n* = 30), and never captured more than once a week. Every time an individual kestrel was captured, we measured its body mass. GPS-dataloggers were programmed to collect data only during daylight hours (5 to 20 h UTC) and during the breeding season (March – July). GPS devices provided the flight altitude and instantaneous speed for each location. We removed the harnesses from the kestrels at the end of each breeding season. The tracking data can be consulted on Movebank (www.movebank.org) [41].

### Foraging movement variables

GPS locations were graphically explored using GIS (ArcGIS 10, ESRI, Redlands, California, U.S.A.) to identify individual foraging trips. We use the term foraging trip to refer to a set of consecutive locations of an individual kestrel that start from the breeding colony and extend beyond 300 m and in which we are able to identify a foraging event (mostly clumped locations at low altitude above the ground with highly variable instantaneous speed). Incomplete foraging trips, i.e. trips in which departure from or arrival at the colony or roost was not recorded by the GPS were excluded from statistical analyses.

For every lesser kestrel foraging trip we calculated: (1) duration, as the time difference between leaving and returning to the colony or roost; (2) distance, as the accumulated distance traveled between consecutive spatial locations along the trip; and (3) the maximum distance from the colony reached along the trip. For every complete day of tracking, which are those dates and individuals in which we obtained tracking data from sunrise to sunset, we calculated: (1) daily distance, as the accumulated distance traveled between consecutive spatial locations recorded through day; (2) the number of foraging trips performed along the day; and (3) daily colony attendance, as the percentage of daytime that individual spent at the colony. We considered that individuals were at the colony when spatial locations were registered within a 50 m-buffer from the colony. We calculated day length as the difference between sunrise and sunset times provided by Ministerio de Fomento of Spain (http://www.fomento.es).

Every foraging trip and complete day of tracking was assigned to one of the phenological periods into which we divide the breeding season of each breeding pair using the laying and hatching date at their nest: establishment (from the arriving at the breeding colony after spring migration until courtship), courtship (21 days before laying the first egg, see [35]), incubation (between laying and hatching of the first egg), and nestling (from hatching of the first egg until fledging of the last chick). Nest-boxes installed in both colonies are equipped with analogue video cameras (Videcon, model KPC-EX500B) that record 10-s video samples when activated by movement inside the nest-boxes. Individual laying and hatching dates were determined using these video samples. In addition, media samples also provided us with the brood size and also chick age at any time during the nestling period.

### Foraging habitat use

To study sexual differences in foraging habitat uses by lesser kestrels, we first filtered the positions of the trip corresponding to a foraging event. Then we randomly selected one GPS position per foraging event. In the field, we located the coordinates of these positions using a hand-held GPS (model GPSmap 60, Garmin). And finally, we recordered the predominant habitat type within a 50-m buffer around the positions. The habitat was categorized into nine different types: cereal (mainly non-irrigated wheat), stubble (harvested cereal), sunflower, seedlings (sunflower and cotton crops when vegetation height was lower than 50 cm), vineyards, tree groves (fruit tree and olive groves), pastures (non-arable lands), ploughed (ploughed and sowed fields), and others (less-used habitats: alfalfa, beetroot, chickpea, cotton, garlic, maize, potatoes, and rice). Both sunflower and cotton plants may grow more than 1 m throughout the breeding season, which might provide substantial differences at the microhabitat level. We therefore consider seedlings as a different category from the full grown plants (see [42]). When different foraging events from an individual kestrel within the same GPS deployment (a time window of 1 week as individuals were never recaptured more than once per week) were coincident on the same location, we considered them as a single foraging location in the analyses. Field visits were carried out 7–15 days after the kestrels had been foraging in the selected location, because locations had to be downloaded and birds were recaptured weekly. Field visits extended over three out of the four lesser kestrel breeding seasons included in the study (years 2012–2014).

### Hunting strategy

We also studied sexual differences in the lesser kestrel’s hunting strategy. As mentioned in the background section, lesser kestrels can capture prey either by hovering flights (an active hunting strategy in which kestrels stay suspended in the air by flapping their wings) or by perch-hunting (a passive sit-and-wait hunting strategy from an elevated position) along foraging trips [30]. Using tri-axial accelerometry, we found that 99% of hovering flights last less than 30 s (*N* = 4933 hovering bouts, *authors unpub. data,* but see [43]), so they can be only identified from 1-s GPS data. In contrast, perching bouts can be also identified at lower GPS sampling frequencies since tri-axial accelerometry reveals that more than 40% of perching bouts last more than 1 min (*N* = 2798 perching bouts, *authors unpub. data,* but see [43]). Therefore, we focused the study of the lesser kestrel foraging strategy on the relative use of perch-hunting during foraging trips throughout the breeding season. We considered a perching bout as a sequence of GPS locations in which the distance between consecutive locations was below 1 m (1-s GPS sampling frequency), 5 m (1-min frequency), 15 m (3-min frequency), 25 m (5-min frequency) or 50 m (10-min frequency). We increased the buffer with sampling frequency because GPS spatial accuracy decreases as GPS sampling frequency decreases [44]. We then calculated the total time perching per foraging trip as the sum of the duration of all perching bouts per foraging trip. To be conservative, we discarded from the statistical analyses those perching bouts that lasted less than 30 s as they could also be hovering bouts.

### Statistical analyses

To test hypotheses 1 and 2 and evaluate the effect of either role specialization or sexual dimorphism on foraging movement patterns throughout the breeding season, we fitted Generalized Linear Mixed Models (GLMMs) to foraging movement variables at the daily level (distance traveled, number of foraging trips, colony attendance), and at foraging trip level (duration, distance, maximum distance). To test hypothesis 3, that role-specialization and not size dimorphism is the best explanation for observed differences, we always tested the interaction between sex (categorical predictor with 2 levels: female and male) and phenological period (categorical predictor with 4 levels: establishment, courtship, incubation, and nestling). We always incorporated GPS sampling frequency as a correction factor with 5 levels (1-s, 1-, 3-, 5- and 10-min frequency) for foraging trip variables, or 3 levels: (1-, 3- and 5-min frequency) for variables at the daily level, because GPS sampling frequency influences the estimation of movement variables, especially those related to distance. Given that it could be non-linear, to test if parental investment increases along the nestling period (hypothesis 8), we used Generalized Additive Mixed Models (GAMMs) to predict kestrel foraging movement variables at the daily level. In order to test hypotheses 1, 2 and 3 we included the interaction between parental sex and eldest chick age (continuous predictor) in these models. We also included brood size at the date each variable was registered as a continuous predictor because brood size could influence energy demand (and GPS sampling frequency was included as a correction factor with 3 levels). To study adult kestrel breeders’ body mass evolution along the breeding season and to determine whether the increase in energy demand influences their body weight along the breeding period we fitted a GAMM model to the kestrel body mass with the interaction between sex and day-of-year as predictors. In order to assess the temporal changes in the use of perch-hunting by both sexes throughout the breeding season we also fitted GLMMs to total perching time per foraging trip and to the presence of perch-hunting in the foraging trip (a binary response variable, 0 = “no perching bouts”, 1 = “at least one perching bout”). Individual identity, year, and breeding colony were included as random factors in all models. The complete list of models fitted at each level and predictors used are detailed in Table 1. Some response variables were transformed to obtain a proper fit for the models. Arcsine-square-root transformation was applied to percentage variables and logarithmic transformation was applied to multiplicative variables.

**Table 1:** Summary of statistical analyses of lesser kestrel foraging movement variables

Level of Analyses	Response Variable	Predictors Tested	Correction Factor	Random Factors	Error Distribution/Link Function
Daily	Distance traveled	Sex * Phenological period,	GPS sampling frequency	Individual, Year, Breeding colony	Gaussian/Identity
	# Foraging trips				Poisson/Logarithmic
	Colony attendance (arcsine-square-root)				Gaussian/Identity
Nestling period (daily)	Distance traveled	Sex * Eldest chick age, Brood size			Gaussian/Identity
	# Foraging trips				Poisson/Logarithmic
	Colony attendance (arcsine-square-root)				Gaussian/Identity
Foraging Trip	Duration (logarithm)	Sex * Phenological period,			Gaussian/Identity
	Distance (logarithm)				Gaussian/Identity
	Maximum distance (logarithm)				Gaussian/Identity
	Probability of perching bout				Binomial/Logit
	Total perching time (logarithm)				Gaussian/Identity
Body condition	Body mass	Sex * Day-of-year			Gaussian/Identity

Transformation of response variables is shown in brackets

We applied penalized smoothing splines to eldest chick age and to day-of-year in the GAMMs. The degrees of freedom of the smoothing function were automatically selected using restricted maximum likelihood (REML) [45]. We followed the Akaike’s Information Criterion (AIC) and AIC weights for model selection [46]. As the best GAMMs fitted to all three daily foraging variables calculated at the nestling period were those including a linear effect of eldest chick age, we simplified the models by fitting a GLMM to all variables with the same error distribution and link function as in the GAMMs. They included the same fixed and random factors used in the GAMMs. We fitted the GLMMs using a backward-stepwise procedure to remove the non-significant predictors, thereby maintaining only the significant ones. The significance of the predictors was tested using likelihood ratio tests comparing the model with and without the predictor. We evaluated statistical significance between levels of the categorical predictors of the models by applying Holm’s correction for multiple comparisons [47].

Statistical analyses were performed using the R-software 3.1.1 [48] fitting GAMMs and GLMMs using “mgcv” [49] and “lme4” [50] packages, respectively. Post-hoc comparisons between categorical predictor levels were assessed using “phia” package [51]. Statistically significant differences with *p*-value < 0.05 are referred to as significant. Results are shown as mean ± standard deviation. The parameters of the models fitted to transformed response variables were presented on the original scale after back-transforming them in order to better understand the effect of the predictors on these response variables.

## Results

### Daily level

We obtained 244 complete days of tracking, a mean of 8.41 ± 6.39 per individual lesser kestrel (Table 2). We summarize descriptive statistics of foraging movement variables at the daily level in Additional file 1. As predicted in hypothesis 1, we found sexual differences in all movement variables tested (Tables 3 and 4). Contrary to hypothesis 2 (dimorphism) and in support of hypothesis 3 (role specialization), we found a significant interaction between sex and phenological period on all three kestrel movement variables measured at the daily level (Table 3, Additional file 2). Individuals flew on average daily distances of 97.82 ± 46.22 km with a mean of 6.67 ± 6.14 foraging trips per day during the breeding season. Contrary to hypothesis 2, we did not find overall significant differences between sexes in daily distance traveled, although males flew larger daily distances than females in the nestling period. In support of hypothesis 5, whereby males are the provisioning sex, we found significant differences between sexes in the daily number of foraging trips, with females performing fewer foraging trips per day than males. However, both sexes performed similar daily number of foraging trips during the establishment period as predicted by hypothesis 4 (no difference when role specialization is low). Individuals stayed at the colony on average 19.41 ± 12.94% of daylight hours during the breeding season, with no overall significant differences between sexes, although females stayed longer than males at the colony during the nestling period as predicted by hypothesis 6, whereby females are devoted to defensive tasks (Table 3, Additional file 2).

**Table 2:** Number of individual lesser kestrels tracked, foraging trips, and complete days of tracking used in the statistical analyses separated by sex and phenological period

Level of Analyses	Establishment		Courtship		Incubation		Nestling	
	Female	Male	Female	Male	Female	Male	Female	Male
# Tracked Individuals	6	6	7	10	11	12	11	14
# Complete Days	23	26	28	34	21	28	28	56
# Foraging Trips	82	108	78	285	69	170	332	1047

**Table 3:** Chi-square statistic and statistical significance of the predictors included in the GLMMs fitted to lesser kestrel movement variables at the daily level determined by likelihood ratio tests

		Predictors						
Level of Analyses	Response Variables	Sex * Phenological Period	Sex	Phenological Period	GPS Sampling Frequency	Sex * Eldest Chick Age	Eldest Chick Age	Brood Size
Daily	Distance Traveled	18.92 ***	2.87	39.96 ***	46.29 ***	**-**	**-**	**-**
	# Foraging Trips	14.95 **	17.98 ***	291.95 ***	3.42	**-**	**-**	**-**
	Colony Attendance	24.84 ***	3.14	52.03 ***	3.08	**-**	**-**	**-**
Nestling period (daily)	Distance Traveled	-	2.49	-	5.75	16.06 ***	1.24	0.72
	# Foraging Trips	-	8.84 **	-	4.18	5.43 *	5.47 *	1.39
	Colony Attendance	-	3.90 *	-	10.30 **	15.86 ***	20.28 ***	0.88

Statistically significant predictors are shown in bold: * *p* < 0.5, ** *p* < 0.01, *** *p* < 0.001. – indicates predictor not tested because it was not applicable. Sample size = 244 complete days and 84 complete days at the nestling period

**Table 4:** Chi-square statistic and statistical significance of the predictors included in the GLMMs fitted to lesser kestrel movement variables at the foraging trip level determined by likelihood ratio tests

		Predictors			
Level of Analyses	Response Variables	Sex * Phenological Period	Sex	Phenological Period	GPS Sampling Frequency
Foraging Trip	Duration	29.90 ***	14.02 ***	226.69 ***	23.44 ***
	Distance	6.37	9.38 **	107.69 ***	24.40 ***
	Maximum Distance	4.20	4.95 *	62.33 ***	7.49
	Probability of Perching Bout	30.59 ***	6.35 **	81.73 ***	0.42
	Total Perching Time	10.48 **	17.44 ***	67.50 ***	99.91 ***

Statistically significant predictors are shown in bold: * *p* < 0.5, ** *p* < 0.01, *** *p* < 0.001. Sample size = 2171 foraging trips

**Fig. 1:**
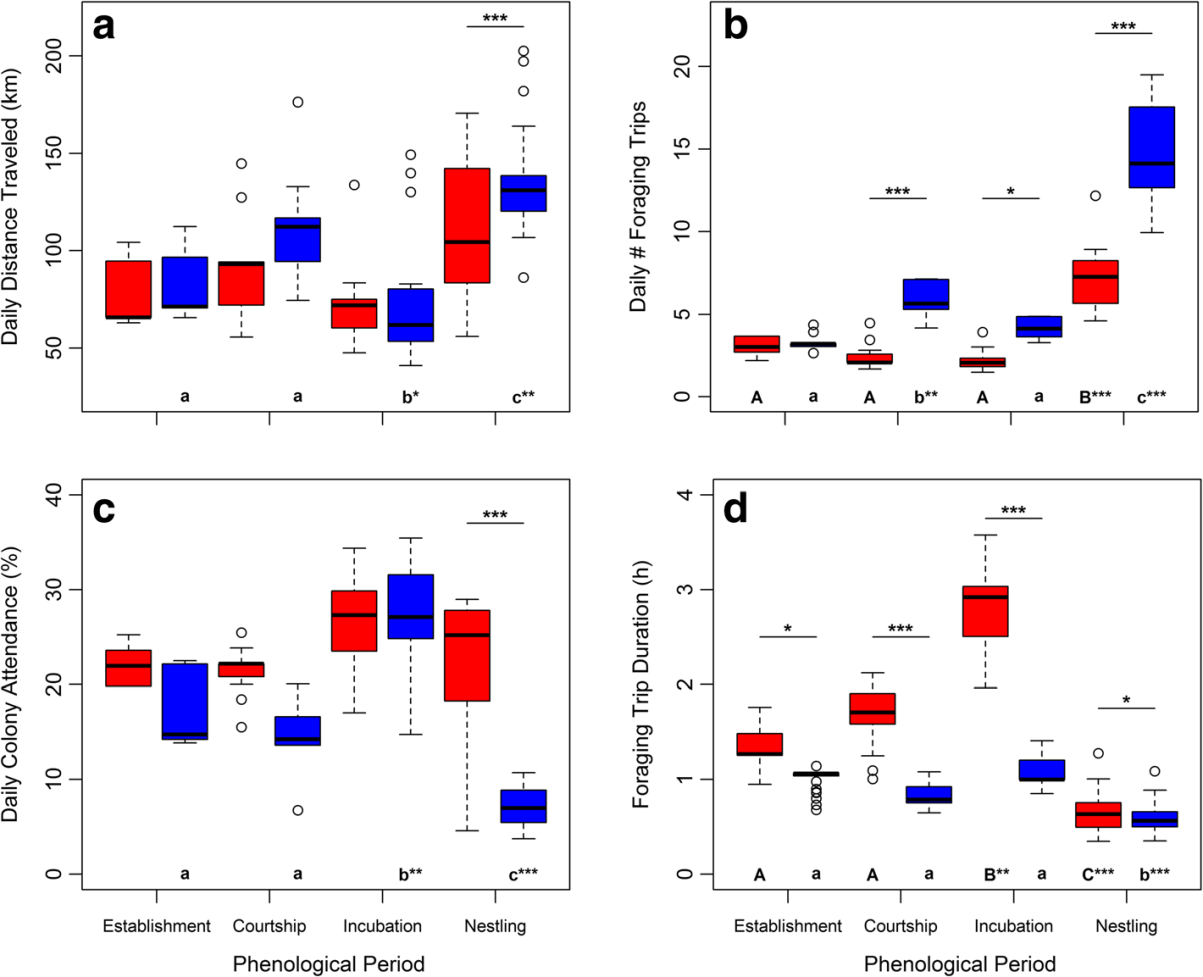
Effect of the interaction between sex and phenological period on lesser kestrel daily distance traveled (**a**), daily number of foraging trips (**b**), daily colony attendance (**c**), and foraging trip duration (**d**) as predicted by GLMMs. Colors indicate kestrel sex: female in red and male in blue. Significance of post-hoc comparison between sexes within phenological periods is indicated above the bar pairs. Significance of post-hoc comparison between phenological periods within sexes is indicated under the bars: values not sharing a common letter are significantly different, either uppercase letters for females or lowercase letters for males. *P*-values are indicated: < 0.5 (*), < 0.01 (**), and < 0.001 (***). Sample size = 244 complete days and 2171 foraging trips

Post-hoc comparisons revealed intra-sexual differences in daily movement variables throughout the breeding season (Fig. 1). Males traveled a similar daily distance during the establishment and courtship periods but they flew shorter distances during the incubation period and larger distances during the nestling period. Furthermore, they increased the daily number of foraging trips from the establishment to the courtship period, which then decreased during the incubation period and increased again towards the nestling period. As a consequence of this foraging investment by males, they stayed at the colony a similar percentage of daylight hours during the establishment and courtship periods but stayed longer during the incubation period and less time during the nestling period. These results were expected for males in hypothesis 7 (parental investment). Females traveled similar daily distance and showed similar daily colony attendance across all phenological periods throughout the breeding season. In addition, females completed similar number of foraging trips per day throughout the breeding season except during the nestling period when they completed more, supporting hypothesis 7.

### Nestling period (Daily Level)

**Fig. 2:**
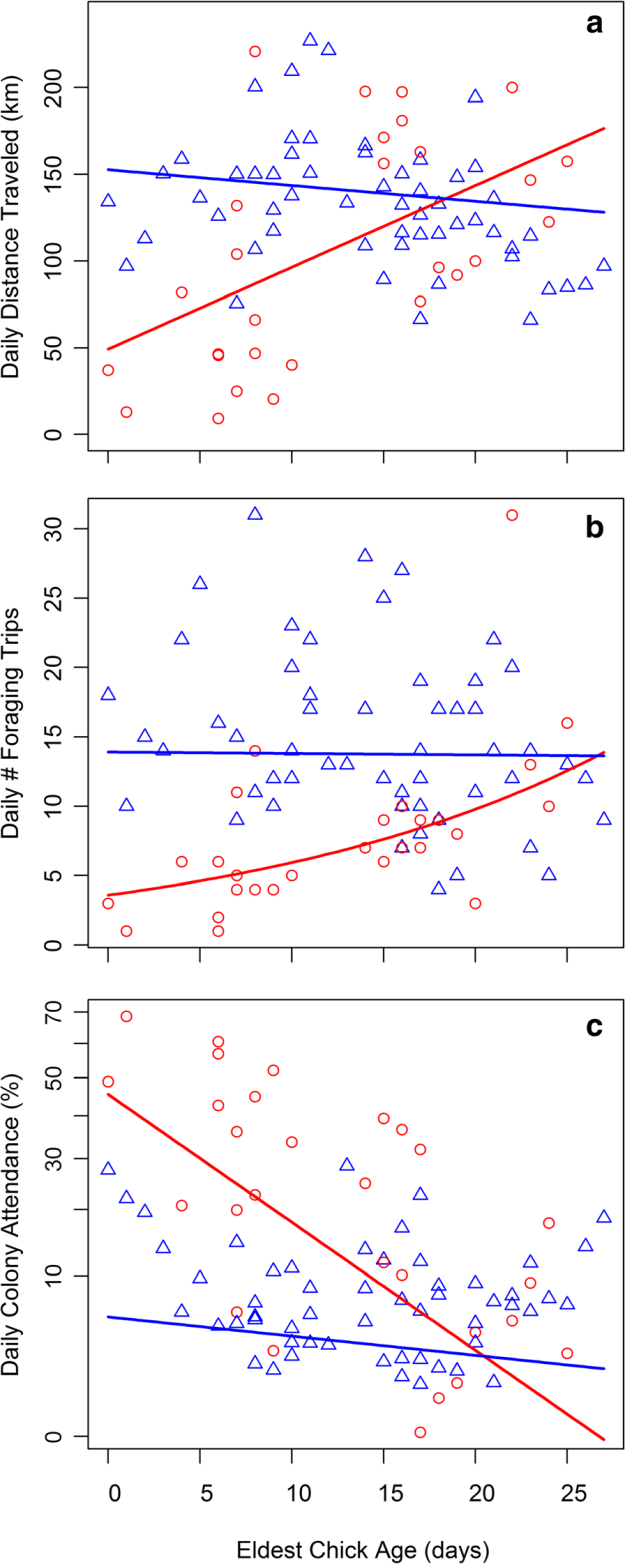
Effect of the interaction between sex and eldest chick age on lesser kestrel daily distance traveled (**a**), daily number of foraging trips (**b**), and daily colony attendance (**c**) during the nestling period predicted by GLMMs. Regression lines are depicted for females (*red circles*, *red line*) and for males (*blue triangles*, *blue line*). Sample size = 84 complete days

We obtained 84 complete days of tracking during the nestling period, a mean of 3.36 ± 3.59 per individual lesser kestrel. We found significant effect of the interaction between parental sex and eldest chick age on the three kestrel foraging movement variables at the daily level, supporting hypothesis 3 (Fig. 2, Table 4, Additional file 3). Males maintained daily distances traveled and performed similar number of foraging trips per day as the chicks grew older, whereas both variables sharply increased in females. This is in partial agreement with hypothesis 8 in which we predicted that both sexes would increase their effort along this period. Males and females reduced daily colony attendance as the nestling period progressed, although the trend was steeper in females. We did not find any significant effect of brood size on these movement variables (Table 3, Additional file 3).

### Foraging trip level

**Fig. 3:**
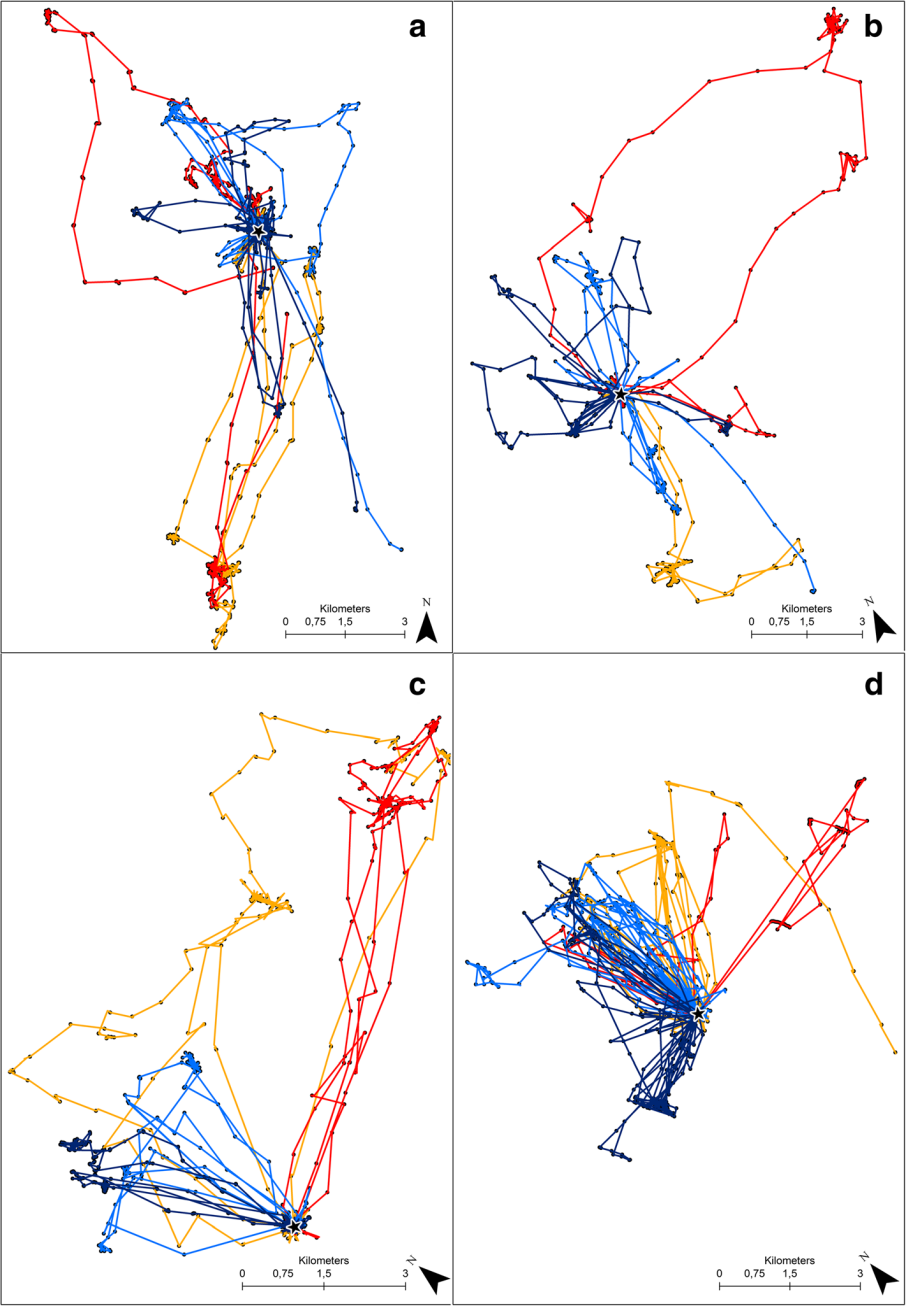
GPS data sampled at 3-min frequency from a complete day of tracking of 4 random individual lesser kestrels in each phenological period: Establishment (**a**), courtship (**b**), incubation (**c**), and nestling (**d**). Colors indicate kestrel females (*red and orange*) and kestrel males (*light and dark blue*). The black star indicates the location of the breeding colony

We identified 2171 complete foraging trips, a mean of 72.37 ± 69.88 per individual lesser kestrel (Table 2). We summarize descriptive statistics of foraging movement variables at the foraging trip level in Additional file 1. Supporting hypotheses 1 and 3, and in disagreement with hypothesis 2, there was a significant interaction between sex and phenological period in the model fitted to foraging trip duration. This indicates a different foraging movement strategy between sexes during the breeding season influenced by reproductive roles (Table 4, Additional file 4). Kestrels performed foraging trips of a mean duration of 1.16 ± 1.28 h throughout the breeding season. We found overall significant differences between sexes, as hypothesis 1 predicts, with females performing longer foraging trips than males, in support of hypothesis 2. Foraging trip duration for males was constant across all phenological periods except in the nestling period when trips were shorter. By contrast, foraging trip duration for females was similar during the establishment and courtship periods, but they became longer during the incubation period and shorter during the nestling period (Fig. 1), supporting hypothesis 3. We did not find any significant interaction between sex and phenological period on foraging trip distance nor on foraging trip maximum distance from the colony, variables for which our hypotheses did not make specific predictions, but we observed significant effects of sex and phenological period on both variables (Table 4, Additional file 4). Individuals flew on average 10.98 ± 11.22 km and reached a mean of 3.68 ± 3.40 km from the colony during each foraging trip throughout the breeding season. Females flew larger distances and also went farther from the colony during their foraging trips compared to males (Fig. 3). But both sexes flew similar distances and reached similar maximum distances from the colony during the establishment, (supporting hypothesis 4) and courtship periods, but both variables increased in the incubation period and decreased during the nestling period (Table 4, Additional file 4).

### Foraging habitat use

**Fig. 4:**
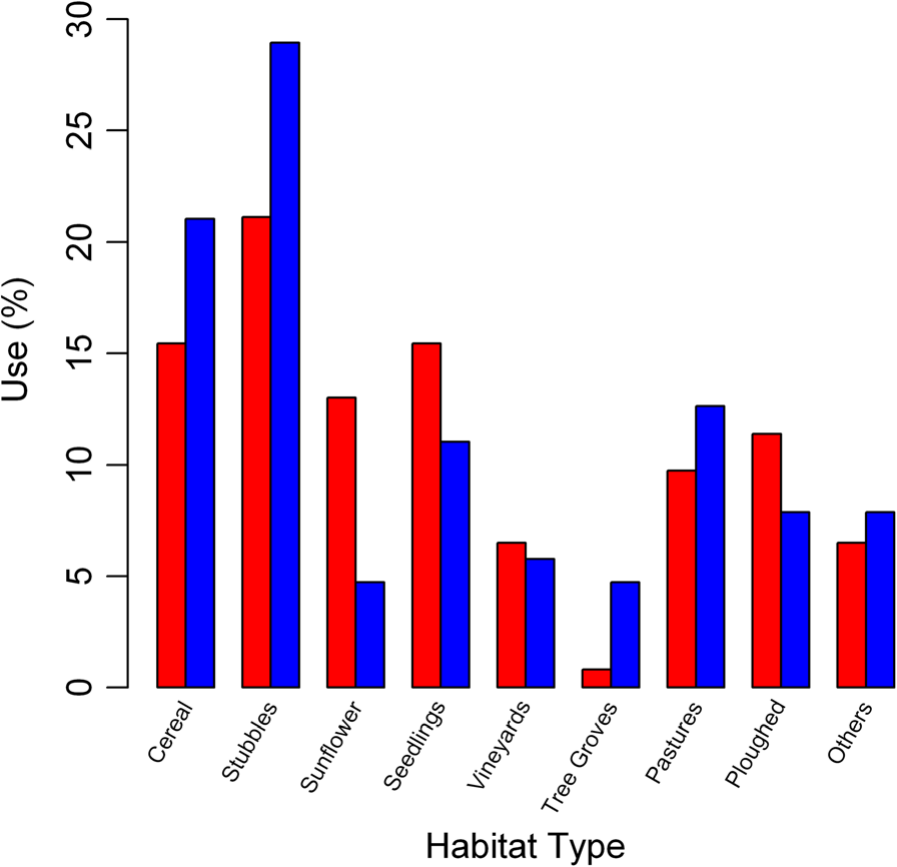
Lesser kestrel percentages of habitats used by each sex for foraging: female in red and male in blue. Sample size = 322 foraging locations

To test if sexual differences in movement strategy could be due to a different habitat selection we gathered information about habitat use by lesser kestrels by visiting 322 foraging locations (a mean of 10.73 ± 9.88 per individual, range 0–34, *n* = 30). We did not find any difference in foraging habitat use between sexes during the breeding season that could justify a different movement strategy (chi-squared test: *χ* ^2^ = 9.49, *p* = 0.30) (Fig. 4). Individuals predominantly used non-irrigated cereals as foraging habitat either when harvested (stubble = 25.05%) or non-harvested (cereal = 18.26%), followed by seedlings (12.42%), pastures (11.18%), ploughed (9.01%), sunflower (7.76%), others (7.14%), vineyards (5.90%) and tree groves (3.10%).

### Perch-hunting strategy

**Fig. 5:**
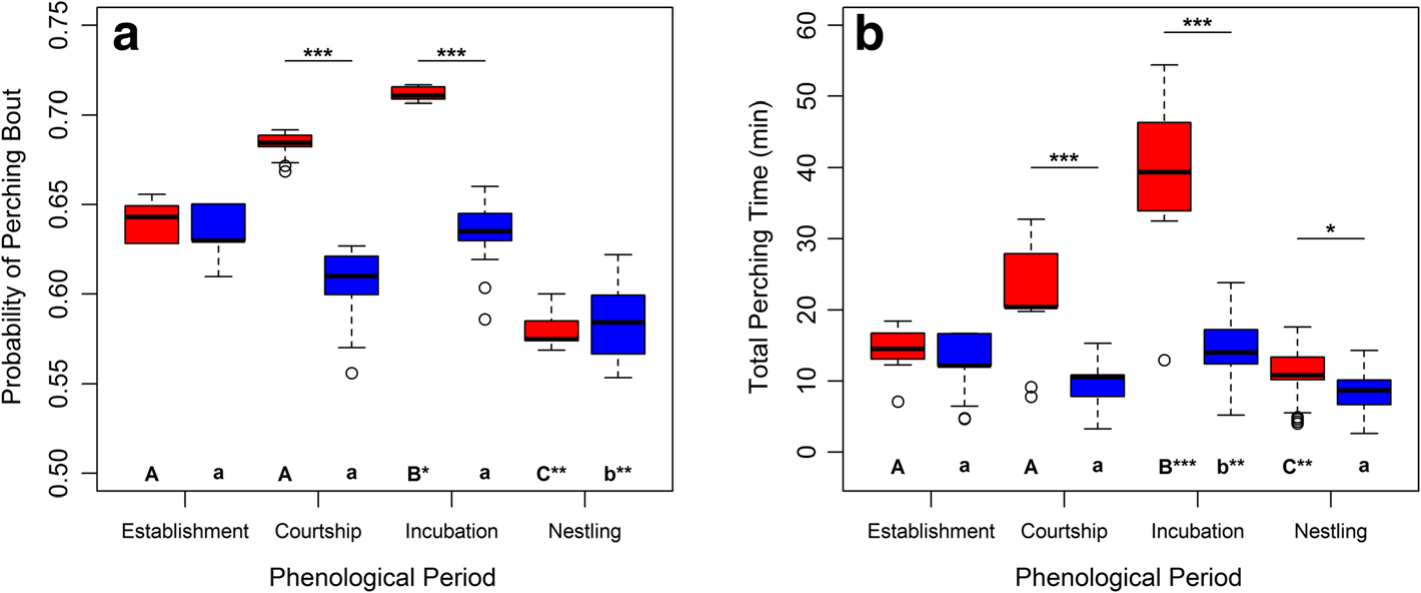
Effect of the interaction between sex and phenological period on the probability of performing a perching bout (**a**) and the total perching time (**b**) during foraging trips predicted by GLMMs. Colors indicate kestrel sex: female in red and male in blue. Significance of post-hoc comparison between sexes within phenological periods is indicated above the bar pairs. Significance of post-hoc comparison between phenological periods within sexes is indicated under the bars: values not sharing a common letter are significantly different, either uppercase letters for females or lowercase letters for males. *P*-values are indicated: < 0.5 (*), < 0.01 (**), and < 0.001 (***). Sample size = 2171 foraging trips

We identified 3271 perching bouts during foraging trips (a mean of 1.51 ± 3.07 bout per foraging trip, range 0–27, *n* = 2171). We had a sample of 2171 foraging trips that were classified as “without perching bouts” (*n* = 1263) or as “with perching bouts” (*n* = 908). In those foraging trips with perching bouts, the total perching time was on average 21.79 ± 28.76 min, (range 0.52–215.00 min per foraging trip). In agreement with hypothesis 3, the best model fitted to the probability of performing a perching bout during foraging trips included the interaction between sex and phenological period (Table 4, Additional file 5). Also the best model fitted to total perching time per foraging trip included the interaction between sex and phenological period (Table 4, Additional file 5). On average, females showed higher probability of performing a perching bout, and they perched longer during foraging trips than males throughout the breeding season. In agreement with hypothesis 4, during the establishment period both sexes showed similar probabilities of performing a perching bout and perched the same amount of time. In the courtship and incubation periods females were more likely to perch, and perched longer, and during the nestling period, both sexes were equally likely to perch but females perched longer (Fig. 5).

### Body condition

**Fig. 6:**
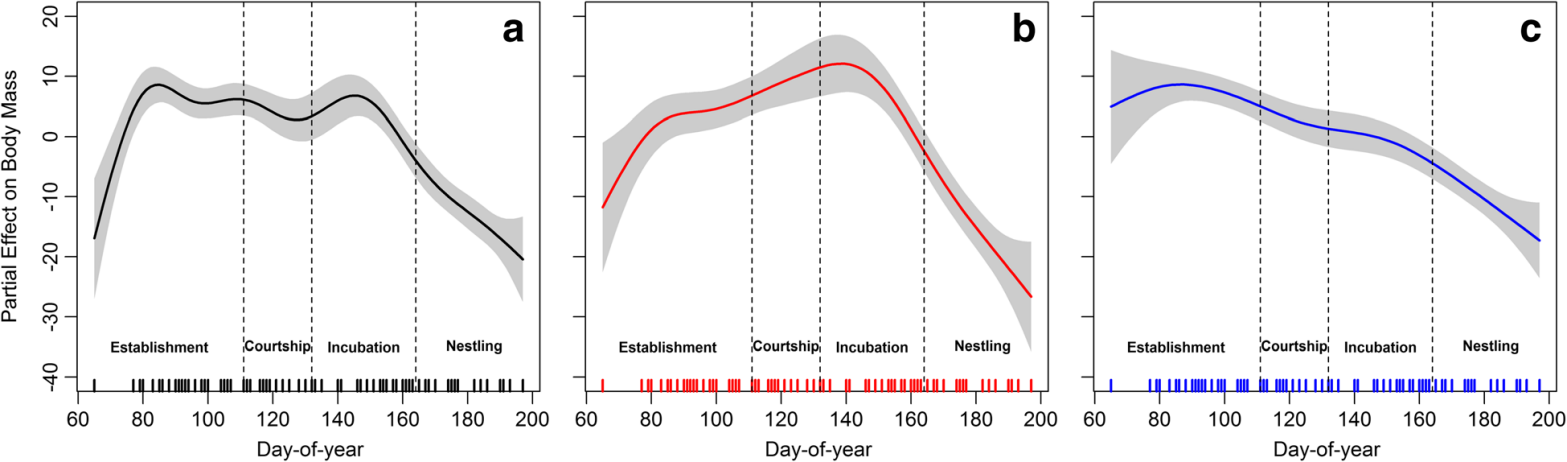
Partial effects of the day-of-year (best GAMM) and the interaction between sex and day-of-year (second best GAMM) on lesser kestrel body mass. A penalized smoothing spline with 7.52° of freedom was adjusted to day-of-year in the best GAMM fitted to lesser kestrel body mass (**a**). Penalized smoothing splines of 3.88 and 5.26° of freedom were adjusted to day-of-year for females (**b**) and males (**c**), respectively, resulted from the second best GAMM fitted to kestrel body mass. Grey shading represents the standard error of the mean effect. The dashed lines show the mean starting days of courtship, incubation and nestling periods. Sample size = 275 individual body masses

We obtained 275 measurements of lesser kestrel body mass (a mean of 7.08 ± 2.87 measurements per year and individual, range 0–11, *n* = 30). The best model fitted to body mass included day-of-year and sex as predictors (Table 5). Males weighted on average 18 g less than females (Model estimate ± standard error =−18.28 ± 3.49 g), in agreement with the already known reversed sexual size dimorphism in this species. Body mass showed a more or less steady trend from the beginning of the breeding season until the incubation period when it rapidly decreased towards the end of the nestling period, as we had predicted if body mass followed the increase in parental investment (Fig. 6). Although the model did not include the interaction between day-of-year and sex, we showed the different evolution of body mass of males and females throughout the breeding season. We do that in order to get a more detailed view of the process, because the difference in body mass evolution between sexes has already been described during the breeding season [52]. Male body mass gradually decreased as the breeding season progressed, whereas female body mass increased from the establishment period to the incubation period and then rapidly decreased towards the end of the breeding season (Fig. 6).

**Table 5:** AIC-based selection of the model fitted to lesser kestrel body mass

Predictors	AIC	ΔAIC	W_i_
Smoothed (Day-of-year)*Sex	2115.84	11.21	0.004
Smoothed (Day-of-year) + Sex	**2104.63**	**Best Model**	**0.996**
Day-of-year*Sex	2165.91	61.28	0.000
Day-of-year + Sex	2159.92	55.29	0.000
Smoothed (Day-of-year)	2126.28	21.65	0.000
Day-of-year	2181.33	76.70	0.000
Sex	2229.88	125.25	0.000

AIC weight for each model proposed is indicated as W_i_. The best model is indicated in bold

## Discussion

The application of tracking technologies has provided researchers with valuable spatiotemporal information of parental behavior beyond the nest. This has broadened our knowledge about avian breeding ecology (e.g., [53, 54]). This paper presents evidence on how role specialization by lesser kestrels during the breeding season is reflected in foraging movement behavior, as we hypothesized in accordance with the general trend of role specialization in raptors. Studies in this field have been conducted mostly using marine birds as models; they therefore constitute the only reference to compare our results although the ecological conditions experienced by kestrels can be very different.

Lesser kestrels arrive at the colony in mid-February after the spring migration, but it is not until mid-April that breeding pairs form and reproduction starts (see [55, 56]). How lesser kestrels spend their time and energy during the establishment period is not yet clear. Kestrels appear to explore the surroundings of the colony presumably to create a cognitive map of foraging areas to be used later during the breeding season [40]. However, the spring migration sharply reduces the individual’s fuel reserves, which could have serious carry-over effects on its fitness [57, 58]. Therefore, lesser kestrels of both sexes probably dedicate most of their effort to self-maintenance during this period in order to recover fuel reserves, as observed in other species [59]. The absence of sexual divergence in the daily foraging movement variables observed during the establishment period suggests that sexual dimorphism in size or color is not the main driver of the differences in movement strategy observed in other periods (Fig. 1). Nevertheless, once the breeding pair is formed (the courtship, incubation and nestling periods), lesser kestrels show sexual differences in foraging movement patterns that supports most of our hypotheses regarding the effect of role specialization.

In many avian species, including the lesser kestrel, males deliver food to their mates during the courtship and incubation periods so as to increase the female body condition to help them cope with the energy demand associated with egg incubation [33, 60–62]. The numerous short foraging trips observed in kestrel males follows this mate-feeding behavior, which is an important parental investment, and is reflected in a gradual decrease in male body mass while females increase weight (Fig. 6). Despite being fed by males, kestrel females also perform a few long foraging trips per day (Fig. 1). As a result, both sexes show a similar level of parental investment in terms of daily distance traveled, which was unexpected since females were supposed to remain at the colony and save energy to deal with the cost of egg laying and incubation. During this time, kestrel females adopt a perch-hunting strategy more often and also perch for longer on foraging trips than males, causing the sexual difference observed in trip duration (Fig. 5). The perch-hunting strategy is less energy-consuming than hovering flights, although it is also less time-efficient in finding prey [63, 64]. Furthermore, lesser kestrels rely heavily on thermal soaring when foraging under suitable atmospheric conditions in order to reduce the energy cost of the trips [31]. Therefore, kestrel females could reduce the energy expenditure of their long foraging trips greatly by adopting low-cost hunting and commuting flight strategies. In addition, the chromatic dimorphism of the lesser kestrel might afford a sex-specific foraging efficiency that could partially explain the sexual preference observed in hunting strategies. This is analogous to the behavior reported in the two color morphs of the black sparrowhawk *Accipiter melanoleucus* in respect of light levels [65]. The brown plumage with black stripes of kestrel females may act as disruptive camouflage over the landscape background when using a perch-hunting strategy but in turn it might also make them more easily detectable by prey against the sky when hovering. On the other hand, the white belly and underwings of males might reduce the contrast against the sky background and consequently make them more difficult to be detected by prey when hovering.

In species with reversed sexual size dimorphism, females are typically entrusted with defensive tasks because their larger size is advantageous when defending the nest or offspring [21]. This would partially explain why kestrel females remain longer at the colony during the courtship period than males (Fig. 1), given that repelling conspecifics from the nest can be important for a colonial species [66]. Furthermore, on the one hand, the large body mass provides females with higher incubatory efficiency and, on the other hand, it allows them to survive longer without eating [67, 68]. Lesser kestrel females share the task of incubation equally with males during the daytime, although they do most of the incubation at night [33, 34]. The unexpected similar daily colony attendances of both sexes observed during this period is the result of tracking individuals only during the daytime, and our underestimating total female colony attendance (Fig. 1). As a consequence of sharing the incubation, females perform even longer foraging trips than during the courtship period as they do not have need to hurry back to the colony because their mate would be incubating the eggs, in a similar manner to that described in some marine birds [69, 70].

Rearing the offspring involves an increase in parental investment for both members of the breeding pair in order to fulfill the chicks’ energy demand [71, 72], as reflected by the steepest negative trends of kestrel body mass observed during the nestling period (Fig. 6). In order to maximize the energy intake rate for the chicks, which is essential for their growth and survival [37], lesser kestrels perform the shortest foraging trip during this period. This subsequently allows them to complete the highest number of foraging trips per day (Fig. 1). Individuals can shorten their foraging trips by reducing the exploratory component of the trips since they would already be familiar with the foraging area and prey availability distribution in the surroundings of the colony [40]. Additionally, individuals could also reduce foraging trip duration by adopting the time-efficient hover-hunting strategy. This is in agreement with the reduction in the use of perch-hunting observed during the nestling period (Fig. 5). Lesser kestrels preferentially use hover-hunting when prey availability is high [43]; they are therefore expected to favor this strategy during the nestling period when there is a peak in the availability of preferred prey (large Orthoptera) [73]. Our findings indicate that kestrels adjust parental investment to the energy demand associated with chick growth. Males maintained constant parental investment during the whole nestling period, whereas females increased it as the nestling period progressed (Fig. 2). Females performed a higher number of foraging trips and traveled larger distances as chick age increases, probably because the provisioning activity of males is insufficient and females help them to deliver food to the nest, as has been described in other species [20, 25, 74–76]. On the other hand, kestrel males drastically decrease colony attendance from the incubation to the nestling period, which is to be expected because of the elevated energy demand associated with feeding the chicks. Meanwhile, females stay at the colony for a similar amount of time in the incubation period and early in the nestling period, but afterwards they stay less time at the colony as the chicks grow older (Figs. 1 and 2). It has been stated that females stay longer at the nest to brood the chicks during the first days after hatching as they still have a low thermoregulation capacity [77]. It has also been proposed that females stay at the nest longer because they have to divide large prey delivered by males to feed the chicks when they are young. Role specialization thus peaks early in the nestling period with males doing all the prey provisioning and females dealing with nest defense, brooding and food division [78, 79]. Consequently, differences in movement strategy are also the greatest.

Our findings indicate a sexual spatial segregation in the lesser kestrel during the breeding season: females consistently fly farther from the colony than males during their foraging trips (Fig. 3). This is likely the cause of the sex-specific differences in home ranges previously described in this species, with females covering larger areas than males [80]. Spatial segregation between sexes has been attributed to a foraging strategy that aims to reduce intraspecific competition [81, 82]. It has been suggested that sex-specific nutritional requirements may lead to a niche division in prey consumption and/or in foraging habitat uses between sexes that would result in a spatial segregation [19, 83, 84]. There is no evidence of a sex-specific variation in diet in the lesser kestrel, except during the courtship period when females consume higher proportion of mole-crickets (*Grillotalpa grillotalpa*) than males [85]. However, we observe a maintained sexual spatial segregation throughout the whole breeding season (Fig. 3); a different diet does not therefore seem to be the cause. We do not detect any sexual difference in habitat uses by the lesser kestrel (Fig. 4). Both sexes preferentially foraged in cereal crops, either harvested or not, in the line with what has been previously described for the species [42, 86–88]; we cannot therefore consider habitat selection a cause for spatial segregation of sexes. Sexual spatial segregation has also been related to sex-biased competition abilities [89]. In species in which sexes differ in size, the larger sex normally outcompetes the smaller one and displaces it to suboptimal foraging areas [70, 90, 91]. From an individual perspective, it would be more advantageous for both lesser kestrel sexes to forage in areas close to the colony because of the smaller costs in energy and time invested in commuting flights [92]. In a scenario of competitive exclusion, the larger kestrel females would forage closer to the colony and would displace the smaller males to areas located farther. Nevertheless, we observe the opposite pattern with the smaller males foraging closer to the colony than the larger females. The fact that the spatial segregation between sexes is smaller during the establishment period than in the following periods leads us to think that it is not caused by a competitive exclusion, and role specialization might be involved. The male, which is the sex responsible for nest provisioning, may forage close to the colony in order to reduce foraging trip duration and consequently maximize prey delivering rate. Meanwhile, females may fly towards foraging areas farther away in order to reduce competition for food with males, which they could do by thermal soaring with low flight cost. This is important since prey depletion in the surroundings of the colony has been reported as a common negative density-dependent effect in colonial species, including the lesser kestrel [31, 93, 94]. Indeed, during the nestling period when availability of preferred prey is highest [73], and both sexes contribute to feed the chicks, kestrels forage closer to the colony than in previous periods (Fig. 3). Our findings suggest that the sexual spatial segregation could be caused by lesser kestrel breeders aiming to increase offspring survival through reducing prey depletion close to the colony and intersexual competition between members of the breeding pair. Therefore, the sexual spatial segregation of the lesser kestrel might well be a result of an adaptive foraging strategy based on role specialization in order to improve breeding success.

## Conclusions

Lesser kestrels show sex-specific differences in foraging movement strategies throughout the breeding period. Both sexes show similar movement patterns early in the breeding season when there is no role specialization. However, as soon as the breeding pair is formed, sexes show distinct foraging movement patterns in accordance with the role specialization of this species during breeding. Males, which are entrusted with food provisioning tasks, perform a higher daily number of foraging trips and fly larger daily accumulated distances than females. In contrast, females tend to stay longer at the colony since they are primarily devoted to defensive tasks, although they also help the males provisioning the chicks when these approach the fledgling stage and demand is highest. The lesser kestrel shows a sexual spatial segregation around the colonies that may result from an adaptive foraging behavior based on role specialization to reduce intersexual competition close to the colony where prey depletion has a negative effect for colonial breeders. This research complements traditional studies on breeding ecology by providing a new perspective on raptor parental behavior away from the nest using the newest tracking technologies. This study also highlights the plasticity of movements shown by a small raptor species in response to temporal dynamic requirements throughout the breeding season.

## Additional files

Additional file 1: Summary of lesser kestrel foraging variables at the daily and foraging trip levels of analyses. Mean ± standard deviation and range (in brackets) are shown per phenological period and sex. Sample size = 244 complete days and 2171 foraging trips. (DOCX 15 kb)

Additional file 2: Parameters (estimate ± standard error) of the GLMMs fitted to kestrel foraging variables at the daily level. Statistically significant variables are shown in bold: * *p* < 0.5, ** *p* < 0.01, *** *p* < 0.001, indicated in the first level of each predictor. Sample size = 244 complete days. (DOCX 15 kb)

Additional file 3: Parameters (estimate ± standard error) on the GLMMs fitted to kestrel foraging variables at the daily level during the nestling period. Statistically significant variables are shown in bold: * *p* < 0.5, ** *p* < 0.01, *** *p* < 0.001, indicated in the first level of each predictor. Sample size = 84 complete days. (DOCX 16 kb)

Additional file 4: Parameters (estimate ± standard error) of the GLMMs fitted to kestrel foraging variables at the foraging trip level. Statistically significant variables are shown in bold: * *p* < 0.5, ** *p* < 0.01, *** *p* < 0.001, indicated in the first level of each predictor. Sample size = 2171 foraging trips. (DOCX 15 kb)

Additional file 5: Estimates (β), standard error (S.E.) and statistical significance of predictors included in the GLMM fitted to the probability of performing at least a perching bout and to the total perching time during lesser kestrel foraging trips. Statistically significant variables are shown in bold: * *p* < 0.5, ** *p* < 0.01, *** *p* < 0.001, indicated in the first level of each predictor. Sample size = 2171 foraging trips. (DOCX 15 kb)
