# Supplementary material for: A few long versus many short foraging trips: different foraging strategies of lesser kestrel sexes during breeding
Source: Mov Ecol. 2017 Apr 25;5:8. doi: 10.1186/s40462-017-0100-6 (PMC5404669; doi:10.1186/s40462-017-0100-6)
Supplement: Supplementary file 1 — Summary of lesser kestrel foraging variables at the daily and foraging trip levels of analyses. Mean ± standard deviation and range (in brackets) are shown per phenological period and sex. Sample size = 244 complete days and 2171 foraging trips. (DOCX 15 kb) [file 40462_2017_100_MOESM1_ESM.docx]

**Additional file 1** Summary of lesser kestrel foraging variables at the daily and foraging trip levels of analyses. Mean ± standard deviation and range (in brackets) are shown per phenological period and sex. Sample size = 244 complete days and 2171 foraging trips.

| **Foraging Variable** | **Establishment** | | **Courtship** | | **Incubation** | | **Nestling** | |
| --- | --- | --- | --- | --- | --- | --- | --- | --- |
|  | **Female** | **Male** | **Female** | **Male** | **Female** | **Male** | **Female** | **Male** |
| Daily distance traveled (km) | 78.83 ± 27.92 (52.97–181.10) | 81.51 ± 36.37 (47.96–239.00) | 85.84 ± 40.89 (45.08–212.00) | 108.40 ± 39.59 (42.76–209.40) | 70.72 ± 12.56 (55.03–104.00) | 70.79 ± 44.36 (22.56–203.90) | 105.30 ± 65.22 (9.20–220.80) | 132.70 ± 36.24 (66.05–226.90) |
| Daily # foraging trips | 3.04 ± 1.49 (1.00–6.00) | 3.15 ± 1.62 (1.00–7-00) | 2.43 ± 1.42 (0.00–7.00) | 6.00 ± 3.88 (1.00–17.00) | 2.29 ± 0.64 (1.00–4.00) | 4.17 ± 2.24 (1.00–9.00) | 7.68 ± 5.94 (1.00–31.00) | 14.79 ± 5.99 (4.00–31.00) |
| Daily colony attendance (%) | 22.32 ± 7.29 (3.94–36.76) | 18.31 ± 7.26 (8.26–36.65) | 22.32 ± 8.20  (11.52–38.78) | 15.54 ± 7.89  (1.35–36.92) | 26.95 ± 7.95  (8.31–37.60) | 30.29 ± 14.68 (1.73–58.26) | 25.44 ± 20.64 (0.01–68.63) | 8.32 ± 6.56 (1.06–28.45) |
| Foraging trip duration (h) | 2.01 ± 1.93 (0.25–9.01) | 1.59 ± 1.67 (0.13–7.67) | 2.51 ± 2.21 (0.25–9.17) | 1.24 ± 1.21 (0.12–7.92) | 3.39 ± 1.70 (0.20–6.94) | 1.57 ± 1.41 (0.15–8.06) | 0.92 ± 0.92 (0.06–6.99) | 0.79 ± 0.75 (0.05–7.01) |
| Foraging trip distance (km) | 15.10 ± 13.69 (1.04–57.50) | 12.77 ± 13.78 (1.01–84.39) | 20.26 ± 20.25 (1.15–85.30) | 11.56 ± 11.15 (0.99–85.85) | 22.81 ± 12.37 (1.27–49.47) | 12.64 ± 11.17 (1.09–50.29) | 11.87 ± 12.39 (0.69–126.10) | 8.29 ± 7.71 (0.90–70.37) |
| Foraging trip maximum distance (km) | 3.91 ± 2.90 (0.47–13.13) | 3.63 ± 3.22 (0.42–23.69) | 5.35 ± 5.55 (0.47–32.23) | 3.75 ± 3.87 (0.45–24.94) | 5.98 ± 3.02 (0.50–14.63) | 4.02 ± 3.24 (0.50–15.13) | 4.30 ± 3.90 (0.34–19.09) | 3.13 ± 2.79 (0.45–19.06) |
