# Supplementary material for: A few long versus many short foraging trips: different foraging strategies of lesser kestrel sexes during breeding
Source: Mov Ecol. 2017 Apr 25;5:8. doi: 10.1186/s40462-017-0100-6 (PMC5404669; doi:10.1186/s40462-017-0100-6)
Supplement: Supplementary file 2 — Parameters (estimate ± standard error) of the GLMMs fitted to kestrel foraging variables at the daily level. Statistically significant variables are shown in bold: * p < 0.5, ** p < 0.01, *** p < 0.001, indicated in the first level of each predictor. Sample size = 244 complete days. (DOCX 15 kb) [file 40462_2017_100_MOESM2_ESM.docx]

**Additional file 2** Parameters (estimate ± standard error) of the GLMMs fitted to kestrel foraging variables at the daily level. Statistically significant variables are shown in bold: * p < 0.5, ** p < 0.01, *** p < 0.001, indicated in the first level of each predictor. Sample size = 244 complete days.

|  |  | **Response Variable** | | |
| --- | --- | --- | --- | --- |
| **Predictor** | **Level** | **Distance (km)** | **# Foraging Trips** | **Colony Attendance (%)** |
| Intercept | (φ) | 98.57 ± 15.76 | 3.33 ± 1.20 | 15.11 ± 0.16 |
| Sex * Phenological Period | Male - Establishment | **-8.49 ± 14.70***** | **-0.17 ± 1.24**** | **0.86 ± 0.24***** |
|  | Female – Courtship | **-10.10 ± 9.55** | **-0.77 ± 1.20** | **0.19 ± 0.11** |
|  | Male – Courtship | **1.59 ± 13.00** | **2.37 ± 1.26** | **-0.60 ± 0.21** |
|  | Female – Incubation | **-29.13 ± 11.29** | **-1.08 ± 1.24** | **7.23 ± 0.16** |
|  | Male – Incubation | **-38.10 ± 14.51** | **0.58 ± 1.30** | **11.99 ± 0.26** |
|  | Female – Nestling | **-20.61 ± 11.84** | **3.66 ± 1.21** | **2.93 ± 0.17** |
|  | Male – Nestling | **26.83 ± 14.41** | **10.78 ± 1.25** | **-8.73 ± 0.26** |
| Sex | Male | 14.87 ± 8.50 | **1.78 ± 1.13***** | -4.76 ± 0.10 |
| Phenological Period | Courtship | **1.80 ± 7.03***** | **0.76 ± 1.11***** | **-0.54 ± 0.06***** |
|  | Incubation | **-26.20 ± 7.50** | **0.003 ± 1.13** | **9.37 ± 0.07** |
|  | Nestling | **17.98 ± 7.31** | **5.53 ± 1.11** | **-6.76 ± 0.07** |
| Sampling Frequency | 1-minute | **77.91 ± 11.50***** | 0.06 ± 1.13 | -4.99 ± 0.15 |
|  | 3-minutes | **24.94 ± 6.08** | 0.49 ± 1.08 | 0.03 ± 0.04 |

(φ) The intercept includes the effect of female sex, establishment period, and 5-minute GPS sampling frequency
