# Supplementary material for: A few long versus many short foraging trips: different foraging strategies of lesser kestrel sexes during breeding
Source: Mov Ecol. 2017 Apr 25;5:8. doi: 10.1186/s40462-017-0100-6 (PMC5404669; doi:10.1186/s40462-017-0100-6)
Supplement: Supplementary file 3 — Parameters (estimate ± standard error) on the GLMMs fitted to kestrel foraging variables at the daily level during the nestling period. Statistically significant variables are shown in bold: * p < 0.5, ** p < 0.01, *** p < 0.001, indicated in the first level of each predictor. Sample size = 84 complete days. (DOCX 16 kb) [file 40462_2017_100_MOESM3_ESM.docx]

**Additional file 3** Parameters (estimate ± standard error) on the GLMMs fitted to kestrel foraging variables at the daily level during the nestling period. Statistically significant variables are shown in bold: * p < 0.5, ** p < 0.01, *** p < 0.001, indicated in the first level of each predictor. Sample size = 84 complete days.

|  |  | **Response Variable** | | |
| --- | --- | --- | --- | --- |
| **Predictor** | **Level** | **Distance Traveled (km)** | **# Foraging Trips** | **Colony Attendance (%)** |
| Intercept | (φ) | 49.21 ± 19.21 | 3.60 ± 1.25 | 45.55 ± 0.52 |
| Sex * Eldest Chick Age | Female | **4.71 ± 1.11***** | **0.18 ± 1.01*** | **-3.59 ± 0.001***** |
|  | Male | **-0.91 ± 1.31** | **0.003 ± 1.01** | **-0.54 ± 0.002** |
| Sex | Male | 34.69 ± 20.63 | **6.14 ± 1.22**** | **-12.09 ± 0.32*** |
| Eldest Chick Age | - | 0.70 ± 0.65 | **0.05 ± 1.01*** | **-1.31 ± 0.001***** |
| Brood Size | - | 6.03 ± 6.93 | 0.24 ± 1.07 | -2.37 ± 0.03 |
| Sampling Frequency | 1-minute | 34.79 ± 15.60 | -0.52 ± 1.18 | **-4.12 ± 0.25**** |
|  | 3-minutes | 5.30 ± 11.17 | 0.52 ± 1.13 | **19.87 ± 0.19** |

(φ) The intercept includes the effect of female sex, and 5-minute GPS sampling frequency.
