# Supplementary material for: A few long versus many short foraging trips: different foraging strategies of lesser kestrel sexes during breeding
Source: Mov Ecol. 2017 Apr 25;5:8. doi: 10.1186/s40462-017-0100-6 (PMC5404669; doi:10.1186/s40462-017-0100-6)
Supplement: Supplementary file 4 — Parameters (estimate ± standard error) of the GLMMs fitted to kestrel foraging variables at the foraging trip level. Statistically significant variables are shown in bold: * p < 0.5, ** p < 0.01, *** p < 0.001, indicated in the first level of each predictor. Sample size = 2171 foraging trips. (DOCX 15 kb) [file 40462_2017_100_MOESM4_ESM.docx]

**Additional file 4** Parameters (estimate ± standard error) of the GLMMs fitted to kestrel foraging variables at the foraging trip level. Statistically significant variables are shown in bold: * p < 0.5, ** p < 0.01, *** p < 0.001, indicated in the first level of each predictor. Sample size = 2171 foraging trips.

|  |  | **Response Variable** | | |
| --- | --- | --- | --- | --- |
| **Predictor** | **Level** | **Duration (h)** | **Distance (km)** | **Maximum Distance (km)** |
| Intercept | - | 1.03 ± 0.02 | 16.77 ± 1.16 | 3.64 ± 1.13 |
| Sex * Phenological Period | Male - Establishment | **-0.30 ± 0.02***** | -5.28 ± 1.18 | -0.63 ± 1.17 |
|  | Female - Courtship | **0.27 ± 0.02** | 0.77 ± 1.14 | -0.19 ± 1.13 |
|  | Male - Courtship | **-0.38 ± 0.02** | -4.85 ± 1.41 | -0.41 ± 1.16 |
|  | Female - Incubation | **1.01 ± 0.02** | 9.38 ± 1.16 | 1.63 ± 1.14 |
|  | Male - Incubation | **-0.19 ± 0.02** | -2.95 ± 1.17 | -0.04 ± 1.18 |
|  | Female - Nestling | **-0.52 ± 0.02** | -5.90 ± 1.19 | -0.42 ± 1.13 |
|  | Male - Nestling | **-0.61 ± 0.02** | -8.59 ± 1.17 | -1.15 ± 1.16 |
| Sex | Male | **-0.35 ± 0.02***** | **-5.06 ± 1.11**** | **-0.82 ± 1.11*** |
| Phenological Period | Courtship | **-0.001 ± 0.02***** | **0.74 ± 1.08***** | **0.22 ± 1.07***** |
|  | Incubation | **0.42 ± 0.02** | **4.92 ± 1.09** | **1.01 ± 1.08** |
|  | Nestling | **-0.43 ± 0.02** | **-4.82 ± 1.08** | **-0.62 ± 1.07** |
| Sampling Frequency | 1-minute | **0.24 ± 0.02***** | **-2.10 ± 1.13***** | -0.37 ± 1.11 |
|  | 3-minutes | **0.44 ± 0.02** | **-4.18 ± 1.11** | 0.16 ± 1.10 |
|  | 5-minutes | **0.58 ± 0.02** | **-5.73 ± 1.12** | -0.05 ± 1.11 |
|  | 10-minutes | **1.02 ± 0.02** | **-1.16 ± 1.35** | 1.71 ± 1.32 |

(φ) The intercept includes the effect of female sex, establishment period, and 1-second GPS sampling frequency.
