# Supplementary material for: A few long versus many short foraging trips: different foraging strategies of lesser kestrel sexes during breeding
Source: Mov Ecol. 2017 Apr 25;5:8. doi: 10.1186/s40462-017-0100-6 (PMC5404669; doi:10.1186/s40462-017-0100-6)
Supplement: Supplementary file 5 — Estimates (β), standard error (S.E.) and statistical significance of predictors included in the GLMM fitted to the probability of performing at least a perching bout and to the total perching time during lesser kestrel foraging trips. Statistically significant variables are shown in bold: * p < 0.5, ** p < 0.01, *** p < 0.001, indicated in the first level of each predictor. Sample size = 2171 foraging trips. (DOCX 15 kb) [file 40462_2017_100_MOESM5_ESM.docx]

**Additional file 5** Estimates (β), standard error (S.E.) and statistical significance of predictors included in the GLMM fitted to the probability of performing at least a perching bout and to the total perching time during lesser kestrel foraging trips. Statistically significant variables are shown in bold: * p < 0.5, ** p < 0.01, *** p < 0.001, indicated in the first level of each predictor. Sample size = 2171 foraging trips.

| **Predictors** | **Level** | **Probability (%)** | **Perching Time (min)** |
| --- | --- | --- | --- |
| Intercept | (φ) | 60.95 ± 57.81 | 6.96 ± 1.29 |
| **Sex ***  **Phenological Period** | Male - Establishment | **-7.96 ± 59.20***** | **-0.91 ± 1.25**** |
|  | Female - Courtship | **15.96 ± 59.15** | **4.62 ± 1.22** |
|  | Male - Courtship | **-35.10 ± 60.75** | **-1.55 ± 1.29** |
|  | Female - Incubation | **30.11 ± 61.89** | **12.29 ± 1.23** |
|  | Male - Incubation | **-43.66 ± 63.43** | **1.63 ± 1.31** |
|  | Female - Nestling | **-25.78 ± 58.02** | **-0.54 ± 1.22** |
|  | Male - Nestling | **1.28 ± 59.58** | **-2.33 ± 1.28** |
| **Sex** | Male | **-14.33 ± 55.70**** | **-3.27 ± 1.11***** |
| **Phenological Period** | Courtship | **-1.97 ± 55.05***** | **1.38 ± 1.14***** |
|  | Incubation | **13.90 ± 55.65** | **7.74 ± 1.14** |
|  | Nestling | **-18.28 ± 54.81** | **-0.95 ± 1.13** |
| Sampling Frequency | 1-min | -9.61 ± 57.63 | **-1.92 ± 1.24***** |
|  | 3-min | -11.60 ± 56.69 | **5.85 ± 1.21** |
|  | 5-min | -11.07 ± 57.16 | **11.09 ± 1.23** |
|  | 10-min | -16.23 ± 70.79 | **6.69 ± 2.11** |

(φ) The intercept includes the effect of female sex, establishment period, and 1-second GPS sampling frequency.
